# God and the Welfare State - Substitutes or Complements? An Experimental Test of the Effect of Belief in God's Control

**DOI:** 10.1371/journal.pone.0128858

**Published:** 2015-06-10

**Authors:** Gilad Be’ery, Pazit Ben-Nun Bloom

**Affiliations:** 1 Hebrew University of Jerusalem, Jerusalem, Israel; 2 Political Science Department, Hebrew University of Jerusalem, Jerusalem, Israel; University of Perugia, ITALY

## Abstract

Belief in God’s control of the world is common to many of the world’s religions, but there are conflicting predictions regarding its role in shaping attitudes toward the welfare state. While the devout are expected to support pro-social values like helping others, and thus might be supportive of the welfare state, the possibility of taking action is undermined by the belief in God’s absolute control over world affairs and in a morally perfect providence, who is responsible for the fates of individuals. As the literature provides mixed results on this question, this study examines the role of belief in God’s control on welfare attitudes using three priming experiments and two priming tasks, carried out with a design that is both cross-cultural (US vs. Israel) and cross-religious tradition (Judaism vs. Catholicism). We find evidence that, largely, belief in God’s control increases support for income redistribution among Israeli Jews (study 1), American Jews (study 2), and American Catholics (study 3). The findings suggest that the traditional and common political gap between the economic left and the religious, based on the evaluation that religious beliefs lead to conservative economic preferences, may be overstated.

## Introduction

*Rabbi Meir used to say*:

*The critic [of Judaism] may bring against you the argument*, *“If your God loves the poor*, *why does he not support them*?*”*

*If so*, *answer him*, *“So that through them we may be saved from the punishment of Gehinnom*.*”*

*—Babylonian Talmud*, *Tractate Baba Bathra*, *Folio 10a*, *Soncino Edition (translated)*

Rabbi Meir, a Jewish sage of the second century (A.D.), here presents an imagined argument with “the critic,” regarding the theological basis of the religious commandment of charity. In a world controlled by God, an act of charity could be interpreted as an act of heresy. On the one hand, according to the major religious traditions centered around the idea of God, He expects the devout to strive for moral excellence, and to actively build a morally decent society. On the other hand, and according to the same traditions, God is a key actor in world affairs, with His morally perfect providence being responsible for the fate of individuals. The belief in God’s control thus leads to antithetical conclusions: human action is viewed as very important, while at the same time it is undermined by the belief in the absolute control of God over world affairs.

This seemingly inherent contradiction within religious belief gives rise to contradictory expectations with regard to religious support for political institutions in general and the welfare state in particular. Emerging literature in social and political psychology has shown that belief in a controlling God can undermine support for political institutions [1–3]. It is suggested that belief in God’s control of individuals’ life events facilitates coping with the psychological burdens caused by adverse life events, which constitutes a substantial

overlap with the societal function of the welfare state [4,5]. At the same time, the belief in God’s control strengthens individuals’ confidence in the justice of “natural” economic market outcomes–a confidence that weakens the moral logic of the welfare-state [6]. According to this understanding, God and earthly political institutions such as the welfare state function as substitutes for one another.

Yet, belief in a controlling God can also be seen as complementary to earthly political institutions, and as increasing support of them. A substantial body of work shows that religion cultivates self-transcendence, compassion, and pro-sociality [7], binds people together [8], and increases the psychological need for an external source of security [9]. These are thought to result in increased support for a welfare system.

Indeed, recent research shows mixed results with regard to the effect of religion on attitudes toward the welfare state [10–12]. These results are in step with the accumulating evidence on the multi-dimensionality of religiosity and the differential effects of different dimensions on social and political attitudes (e.g., [13]).

To examine whether the belief in God’s control (BGC) is related to increased or decreased support for the political institution of the welfare state, we conducted an experiment priming belief in God’s control among Israeli Jews. Since welfare state attitudes (WSA) were found to be affected by context effects [14], we repeat the experiment among Jews across two political contexts with a very different welfare system and different societal values (the US and Israel). Given that the psychological mechanisms underlying the effect of religion on moral behavior sometimes differ among religions [15], we replicate the experiment across two religious traditions (Catholicism and Judaism) in the United States. The key finding that emerged from this study is that belief in God’s control is related to increased support for welfare. This effect is largely robust across the two religious traditions and the two political contexts.

### Religion and prosociality

The vast majority of world religions emphasize pro-social values in their religious teachings: helping others, altruism, and virtue [16–18]. Furthermore, as religious institutions have historically played a significant role in the provision of welfare and are known for evoking charity and compassion for the weak, religion is known as one of the best predictors of philanthropy and charity (for a literature review, see [7]).

Indeed, there is a growing body of research demonstrating a connection between religiosity and helping behavior [19]. For example, studies show associations between self-reported measures of religiosity and such pro-social characteristics as benevolence [20] and forgiveness [21]. Frequency of church (or synagogue) attendance and religious identification have been found to be positively correlated with philanthropy in both observational studies (e.g., [16]) and experimental studies in which the participants have an opportunity to donate in varying contexts (e.g., [22]). However, recent reviews of accumulated literature argue that thus far researchers have been able to show a complex and only conditional relationship between religion and prosociality [23–25]. There are three important reservations raised in those reviews. Firstly, it is shown that it is not religion in general that is associated with prosocial tendencies but rather only specific dimensions or kinds of religiosity [10]. Secondly, it was argued that religiosity strengthens only certain kinds of prosociality. Specifically, it was asserted that it fosters only in-group solidarity but not universal prosociality [19,26]. Thirdly, the direction of causality was questioned, since cooperative individuals may be more attracted to religion rather than the other way around [23]. It was thus concluded that the only inference that could be derived from the extant literature is that "some forms of religion are prosocial in some situations for some groups” ([23], p. 13).

The complexity of the religion-prosociality nexus calls for a more nuanced research framework for studying it. The concepts of religion and prosociality should be decomposed and each of their specific dimensions examined separately. Indeed, the current research is part of this endeavor: it does not address religion as a unidimensional, coherent whole. In accordance with the multidimensional approach to religion [10,13], we explicitly focus on a particular dimension of religiosity (belief), and even more specifically on belief in God’s control. Additionally, we do not check for the effect of religion on prosociality in general. Rather, we limit ourselves to a specific method of helping others, namely, via the welfare state. Indeed, the welfare state is usually universal in character, applying to all, which makes it useful for ascertaining the limits of religious prosociality. Lastly, this study does not suffer from the reverse causality problem, as its experimental character makes it possible to control for the order of cause and effect.

### Religion and support for the welfare state

Assuming religion increases prosociality under some circumstances, does this effect translate into pro-welfare tendencies? Research on the relationship between religiosity and welfare state attitudes has yielded mixed results. While religiosity seems to impact WSA independently of political context, the direction of the effect varies between contexts, with a strengthening (positive) effect in some countries and a weakening (negative) one in others. In their cross-sectional analysis, Pittau et al. [12] show that while in the US higher worship house attendance is associated with weaker support for redistribution, an opposite association is found in Europe. Furthermore, diversity is also found among European countries. In some European countries (e.g., Germany, Portugal, the UK, France, Greece, and Sweden) religious attendance is negatively associated with redistribution support, while religious people who live in Poland, the Czech Republic, Slovakia, Finland, Austria, Belgium, or the Netherlands tend to be more supportive. In addition, different dimensions of religiosity were found to have opposite relationships to pro-social behavior [27], and with support for welfare policies more specifically [10]. These findings suggest that in general the mechanisms relating religion to welfare attitudes are complex.

In this study, we explore conflicting theoretical expectations concerning the effect of a specific religious belief: belief in God’s control over the world (BGC). As one of the most common beliefs in the world’s religions [3,28], BGC could be considered a good starting point for exploring the effects of religious belief in general. A recent paper by Pitlik and Kouba [9] (described in more detail below) shows that perceived degree of control over one’s life is an important predictor of an individual’s welfare attitudes–more important than other predictors surveyed in the extant literature, such as interpersonal trust. As a result, the authors designate religiosity, with its common (but not universal) belief that God’s control constrains an individual’s ability to shape his or her own fate, as a worthwhile subject for inquiry exploring its effects on welfare attitudes [9]. Further, the effects of belief in God’s control on the endorsement of political institutions has recently received attention in experimental social psychology [1–3,28]. As will be described in detail below, these studies inform expectations regarding the effect of this belief on welfare attitudes.

### The weakening hypothesis: God and the government as substitutes

According to the “substitutability argument” postulated in recent literature [4,5], religion acts as a substitute for state-provided insurance against adverse life events, thus reducing the demand for redistribution as a collective insurance device.

Religious belief is theorized as functioning as a substitute for state welfare in two ways. First, some common themes in the world’s major religions make the psychic costs of adverse life events more bearable [4]. Recent research has generalized this assertion, postulating that God and government answer the same psychological need–the need for external control. Both a strong government and BGC preserve a sense of order and structure in the face of a seemingly chaotic universe. As a result, the existence of one–God or government–compensates for the other. Social psychologists have shown in several experimental studies that manipulating the strength of belief in God or the government alters the belief attributed to the other in the opposite direction [1,2] (the religious community plays an important role as well, providing monetary support to its members when they fall on hard times [4,29]. However, this study focuses on the effects of religious belief). Welfare state institutions should be seen as a particular case of this general assertion–religion satisfies a need for security that the welfare state supplies using redistribution and welfare services. Indeed, in a cross-country comparison, Norris and Ingelhart document an inverse correlation between an efficient welfare state and religiosity. This pattern is further explained by the substitutability between these two kinds of safety net providers, God and the welfare state [5].

Secondly, attributing a dominant role in world affairs to God can be argued to undermine the need for human effort in building political institutions. Laurin et al. [28] show experimentally that priming BGC lowers goal-attainment motivations: when God plays an active role in the world, there is less need for human assertiveness. In a second paper, Laurin et al. [3] generalize this finding to political attitudes, demonstrating that support for state-sponsored judicial punishment decreases when the belief in God’s control is made salient. As per the “substitutability argument,” they suggest that people view God as responsible for distributing punishment, which means that their own actions are less needed, such that experimentally priming with attributions to God of responsibility for distributing punishment has a similar effect to priming for God’s control.

This argument can be imported from the realm of judicial justice to that of economic-social justice. Thus, belief in God’s control over the world–given His omniscience and perfect moral will–suggests that He is the best protector of justice, giving each person his or her due and helping the needy. In comparison, the welfare state seems inadequate and unnecessary. God’s providence substitutes for human-run political and societal institutions. Accordingly, it can be hypothesized that:

H_1_: Increasing salience of belief in God’s control over the world will weaken support for the welfare state and its institutions.

### The strengthening hypothesis: God and the government as complements

Belief in God’s control can also be viewed as increasing support for the welfare state. Using a large pool of cross-sectional surveys, Pitlik and Kouba [9] show that demand for a welfare state is associated with the degree of control one has over his or her life. They argue that individuals who feel that they have no control over their own lives, a belief that means that individual success or failure does not depend on personal effort, may see less justice in “market outcomes” and be more willing to insure themselves using a governmental “security net.” They go on to argue that since belief that one’s fate is determined by God is at the core of many religious traditions, it is probable that religious believers will have a weaker feeling of control over their lives, and make stronger demands upon the welfare state.Although Pitlick and Kouba do not find evidence for stronger welfare attitudes among the religious, the theoretical case they make is strong enough to justify further inquiry. This is especially true since their operationalization of religiosity is limited to a single-item measuring of the subjective importance of religiosity.

Belief in God’s control may also have a positive effect on the “supply side” of the welfare state, that is, on people’s willingness to contribute resources to facilitate its operation. It could be the case that even if God is considered in the religious understanding as running worldly affairs, He is regarded mainly as the regulator of human action, and not as a “competitive provider” who substitutes for state-provided services. In other words, God gives humans freedom of action and His interventions are mainly aimed at rewarding good-doers and punishing wrong-doers. Following this line, a dominant school in social and evolutionary psychology contends that the effect of religious belief on behavior is explained by the *supernatural monitoring* mechanism [17]. The supernatural monitoring hypothesis assumes that ideas of God may promote prosociality by reminding people that they are subject to divine surveillance and its moral judgment. Priming BGC could thus be reasonably assumed to augment this effect–the stronger the cognitive accessibility of the idea of the divine surveillance or intervention of a moralizing God, the greater the expected effect on pro-sociality, and the stronger the support for the welfare state. It could thus be hypothesized that:

H_2_: Increasing salience of belief in God’s control over the world will strengthen support for the welfare state and its institutions.

### Sensitivity to context and religious tradition

The interaction of religious and national cultures has long been recognized in the humanities, and in anthropology, political science, sociology, and more recently psychology (for a review, see [30]). National-political contexts not only affect religious beliefs and norms, but can also moderate the effects of religious culture on individual identity formation. It is worth mentioning a few examples that also have relevance to socio-economic policy. First, Protestantism was found to be associated with individualism and the values of self-expression and prosperity [31], although these values appear to be less important to Protestants in post-Communist national cultures [32]. Secondly, Sasaki and Kim [33] found that individualistic themes were more common in US churches, while communitarian themes were more frequent in Korean churches. As a last example, Stark [34] found that, in Western European nations, religion sustains the moral order only when religious beliefs center on an omnipotent, omniscient, moralizing deity, while this is not the case in East European cultures. Varying national-political contexts (in a relevant manner) is thus often expected to condition effects of religious beliefs on political attitudes. Although directly assessing the effects of context is not the goal of this paper, we were interested in examining the extent to which the effect of BGC on welfare attitudes is sensitive to context, by testing the alternative hypotheses in different political settings and among adherents of different religious traditions (as in [13,35]).

To control for contextual differences, we first replicated our experiment among members of the same religious tradition–Jews–living in substantially different political and cultural contexts; namely, Israel and the United States. Although these countries are home to the world’s two largest Jewish communities in the world, they differ both in the importance of Judaism in the national context and in the prevailing economic ideology. Thus, this comparison allows tapping the robustness of the effect of BGC across political contexts, when holding the religious tradition constant.

Next, while BGC is common to many religious traditions, it cannot be taken for granted that its effect is uniform across different religious traditions [15]. Scholars of religion debate the question whether religion should be studied as a universal phenomenon, above and beyond the differences based on different religious traditions and the political contexts in which they are implicated (e.g., [36]), or whether, to the contrary, these differences are substantial and require sensitivity to particularities (e.g., [37]).

To test for potential differences by religious tradition, we conducted the experiment among both American Catholics and American Jews. Among the Christian traditions, we limited ourselves to adherents of the Catholic tradition because Catholicism has a lower heterogeneity compared to Protestant churches, especially in the US. According to the Pew Research Center’s report, “American Protestantism is very diverse. It encompasses more than a dozen major denominational families, such as Baptists, Methodists, Lutherans and Pentecostals, all with unique beliefs, practices and histories”([38], p. 13). The report also shows substantial differences between these various denominational families in social and political attitudes, including those related to economic policy [38].

We thus collected two samples of American participants, for comparison to the results from Israeli Jews: in one sample, we held constant the religious tradition (American Jews), while in the other, we varied both context and religious tradition, which allowed for detection of differences between religious traditions within the American context.

## Study 1

Priming is typically thought to affect political cognition by increasing the accessibility of particular memory objects [39]. Thus, asking certain questions before the presentation of target items may make some considerations more accessible to respondents [40,41]. This method was used to test the effect of religiosity on political attitudes (e.g., [35]), and the effect of BGC in particular [2,3]. To test for the robustness of this priming method, Study 1 also varies the manipulation technique, utilizing both question-order priming and a priming essay.

### Participants

A total of 113 Israeli Jews participated in the study: 35 political science undergraduates attending The Hebrew University of Jerusalem were recruited for partial course credit and an additional 52 participants were voluntarily recruited utilizing a “snowball” method, with a Qualtrics link distributed to members of the first author’s social network, which mostly consisted of religious individuals who are affiliated with the Israeli version of Modern Orthodox Judaism. In order to add diversity to the religious sample of this study, an additional 26 participants were recruited from the Ultra-Orthodox College in Jerusalem (of these, 16 defined themselves as Ultra-Orthodox). The Ultra-Orthodox (*Haredi*) constitute a Jewish sect that rejects modernity and Zionism. They are different from the Modern Orthodox (or, in Israel, National-Religious) sect, which embraces both modernity and Zionism, at least to some extent [42]. The first two groups participated in a computerized version of the experiment, while the third (an Ultra-Orthodox College in Jerusalem) was implemented in a pen-and-paper version due to limitations posed by the environment of the college (for methodological issues related to the different experimental environment, see the General Discussion section below). Due to the religious character of the hypothesis, we used over-sampling of religious participants, and about three-quarters of the sample defined themselves as religious (n = 78, 69%).

### Ethics statement

This study was approved by the Institutional Review Board of the Political Science department at the Hebrew University of Jerusalem and met all applicable standards for the ethics of experimentation and research integrity. Written informed consent was obtained from participants before they completed the questionnaire. All participants were 18 years of age or older.

### Procedure

After reviewing and signing the consent form, the participants filled out a questionnaire. Participants were randomly allocated to one of three experimental conditions: two priming conditions and a no-prime condition (control). In the first priming condition (question-order prime), the participants were first exposed to a battery of questions regarding God’s control and then asked to answer an array of questions regarding the dependent variables (DVs). In the second priming condition (essay prime), we varied the priming technique, and used a short essay that stresses God’s control of the world. In the control condition, the participants answered the DV items without a prior prompt. After answering the DV items, participants reported additional demographics and controls.

### Measures

#### BGC Primes

1. Question-order Prime. As in Laurin et al. [3], we primed BGC using a battery of three questions measuring participants’ BGC. These questions attempt to render the concept of God’s control cognitively accessible for participants. The three items were: “God, or some type of supreme being, is in control, at least in part, of the events within our universe”; “The events that occur in this world unfold according to God’s, or some other supreme being’s, plan”; and “God, or some other supreme being, makes most events in our world happen” (a scale created by averaging these three items showed very strong internal consistency, with Cronbach's *α* = 0.96.) The respondents were asked to indicate to what extent each of the statements resembled their beliefs, on a five-point scale (1 –“not at all” to 5 –“to a great extent”).
2. Essay Prime. Following previous experimental studies in the field [2,28], participants were exposed to a short essay that makes arguments in favor of BGC, before answering the DV items. The essay stresses the viability of BGC in the face of modern doubts about it. In order to facilitate cognitive processing, participants were also asked to summarize in a few sentences the main point of the essay (see the S1 Appendix for English Translation; Hebrew originals are available upon request).

#### Dependent variables

1. Government Responsibility for Redistribution (*Redistribution)*. We adopted the following item from previous research about attitudes regarding redistribution [10,43,44]: “Please indicate to what extent you agree or disagree with the following statement: ‘The government should take measures to reduce differences in income levels’” (1 –“strongly agree” to 5 –“strongly disagree”; responses were reversed so that higher results would represent higher support for redistribution).
  1. Preferences for Welfare State Expenditure (*Expenditure)*. This scale is based on a module adapted from the ISSP Role of Government Survey (2006; for more information see the survey’s web page: http://www.gesis.org/en/issp/issp-modules-profiles/role-of-government/2006/). The introduction to the module is as follows: “Listed below are various areas of government spending. Please indicate whether you would like to see more or less government spending in each area. Remember that if you indicate ‘much more’, it might require a tax increase to pay for it.” Participants were then asked to report their expenditure preferences in four domains: health, old age benefits, welfare services to the needy, and unemployment benefits, using a 5-point scale (1 –“much less” to 5 –“much more”). A scale for welfare state expenditures was then constructed by averaging responses for the four items (Cronbach's *α* = 0.75).

#### Controls

Religiosity. We have included in our analysis controls for two aspects of religiosity: degree of belief in God’s control over the world (*BGC*, also used as the question-order prime), and a subjective religiosity measure similar to the one used Laurin et al. [3], adjusted to the Israeli context: “How do you consider yourself in terms of religiosity?” (5-point scale, 1 = secular, 5 = Ultra-Orthodox). Demographics. *Age* (in years), *sex* (1 if male), *class* (5 point scale, 1 = low class to 5 = high class)*; political orientation* (1–10, 10 = right wing), *education* (1–6, 1 = less than high school, 6 = BA or more). Table 1 reports the descriptive statistics of the sample.

**Table 1:** Descriptive statistics, Israeli Jews sample (Study 1).

Variable	Mean	SD	Min	Max
Age	26.016	6.094	18	66
Male	0.545	0.500	0	1
Class	3.164	0.819	1	5
Religiosity	3.259	1.432	1	5
Ideology (right)	6.360	2.327	1	10
Education	4.735	1.225	1	6
BGC	3.645	1.402	1	5
Government responsibility for redistribution	3.396	1.162	1	5
Welfare state expenditure	3.486	0.642	1	5

### Results

**Fig 1:**
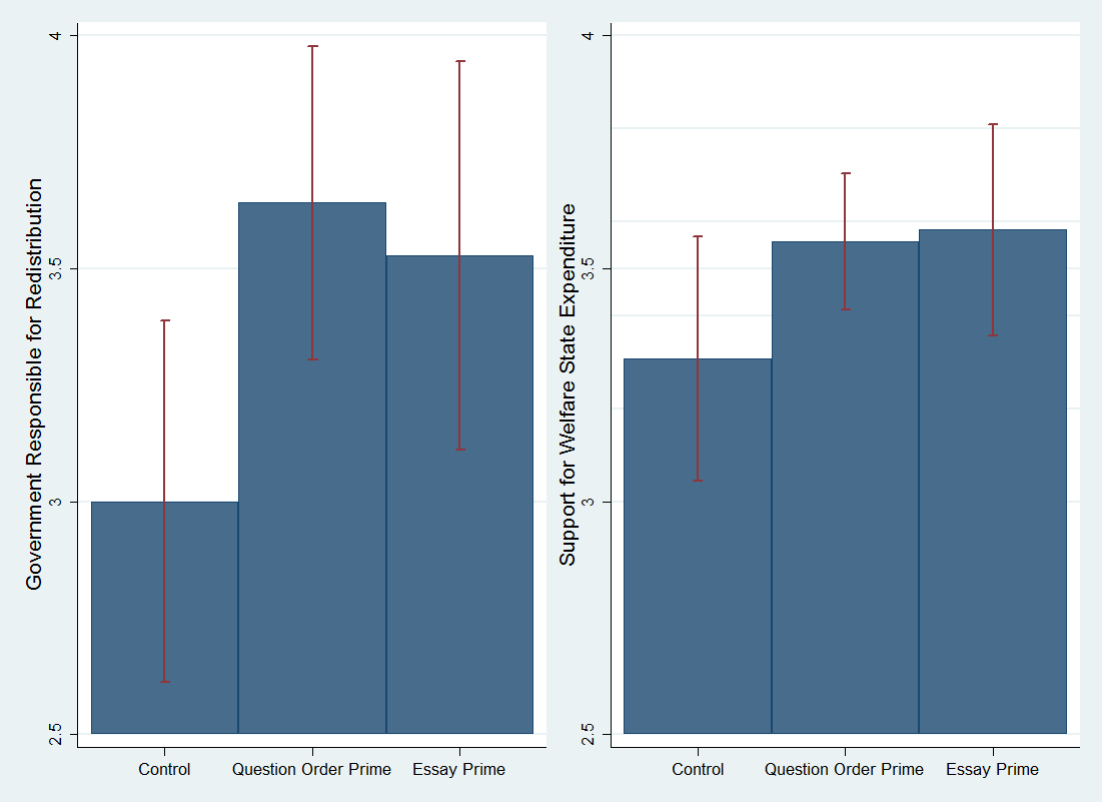
The Effect of the BGC Question Order Prime and the BGC Essay Prime on Support for Redistribution and Support for Welfare State Expenditure among Israeli Jews (Study 1). The graphs include 95% statistical significance bars.

We first examined the means of the two dependent variables, agreement that the government is responsible for redistribution and support for welfare state expenditure, in the control, question order prime of BGC, and essay prime of BGC. Fig 1 plots the difference-of-means test for these variables, presenting the 95% confidence interval bars.

The mean response of welfare attitudes varied systematically among the three groups, with the control condition yielding lower levels of support for welfare compared to the question order prime condition and the essay prime condition (_redistribution_ = 3.00 vs. 3.64 and 3.53 on a 1–5 scale, respectively; _expenditure_ = 3.31 vs. 3.56 and 3.58 on a 1–5 scale, respectively). Looking at the results from a simple ANOVA specifying the two experimental groups with the control condition as baseline, inducing belief in God’s control over the world significantly increases one’s agreement that the government is responsible for redistribution, supporting the strengthening hypothesis (H_2_), and we found this both using the question order task (p_anova_ = .017) and the essay task (p_anova_ = .052). Similarly, both experimental tasks marginally increase support for welfare state expenditure compared to the control condition (p_anova_ = .094 and .070, respectively). Replicating the results using two alternative experimental tasks and two dependent variables is an evidence for their robustness.

Next, the multivariate regression analysis presented in Table 2 tested the effect of the two primes on government responsibility (Model 1) and support for welfare expenditure (Model 3), when holding constant background variables, including measured level of belief in God’s control over the world, religiosity, ideology, age, gender, class and education.

**Table 2:** Effect of BGC on WSA among Israeli Jews (Study 1).

	Government Responsible for Redistribution		Support for Welfare State Expenditure	
	1	2	3	4
BGC Prime 1 –Question Order	0.859**	0.990	0.400*	0.172
	(0.250)	(0.679)	(0.160)	(0.434)
BGC Prime 2 –Essay	0.762**	-0.129	0.429*	-0.339
	(0.265)	(0.766)	(0.180)	(0.445)
Age	-0.0121	-0.00810	0.00702	0.00994+
	(0.0184)	(0.0181)	(0.00600)	(0.00572)
Male	-0.673**	-0.696**	-0.411**	-0.433**
	(0.220)	(0.224)	(0.141)	(0.139)
Class	-0.145	-0.0864	-0.0483	-0.0189
	(0.146)	(0.148)	(0.0711)	(0.0722)
Religiosity	-0.160+	-0.123	-0.0800	-0.0603
	(0.0884)	(0.0905)	(0.0500)	(0.0548)
Ideology (right)	-0.136*	-0.125*	-0.0563	-0.0483
	(0.0600)	(0.0609)	(0.0383)	(0.0384)
Education	-0.00283	-0.0203	-0.0444	-0.0553
	(0.0918)	(0.0938)	(0.0547)	(0.0547)
BGC (measured)	0.136	0.0739	0.0829	-0.00817
	(0.113)	(0.161)	(0.0641)	(0.111)
BGC X Prime 1		-0.0353		0.0639
		(0.175)		(0.108)
BGC X Prime 2		0.251		0.217+
		(0.205)		(0.119)
Constant	4.911**	4.749**	3.913**	4.020**
	(0.821)	(0.977)	(0.373)	(0.526)
Observations	102	102	100	100
R-squared	0.291	0.307	0.259	0.286

Robust standard errors in parentheses

** p<0.01

*p<0.05

+ p<0.1

Holding all else constant, manipulated belief in God’s control significantly increased both measures of support for welfare. Thus, the question order prime increased support for government responsibility and for welfare expenditure by 21% (p_redistribution_ = .001) and 10% (p_expenditure_ = .014) of their range, respectively, and the essay prime increased these two outcome variables by 19% (p_redistribution_ = .005) and 11% (p_expenditure_ = .019) of their range, *ceteris paribus*. As suggested by the effect sizes, the primes had greater impact on the more abstract item, measuring agreement with the general idea that the government should take measures to reduce differences in income levels, than on the more specific measure, tapping the extent to which one is interested in government spending in several areas, given that it may require a tax increase.

Of the control variables, gender has the most consistent effect on welfare attitudes, such that Israeli females are more supportive of welfare policy. This is in line with current work on the gender gap in Israel, suggesting that women’s increased support for welfare may result from differences in values and social roles, as well as enhanced dependency on welfare benefits [45]. Political orientation is associated with decreased attribution of responsibility to the government, which represents the correlation between conservative ideology and weaker support for WSA [46], but this negative association does not reach statistical significance for support for welfare expenditure. Israeli politics is governed by security and foreign policy issues, with economic and social issues typically being of relatively minor concern [47], such that public opinion on these two dimensions is only weakly related [48]. Insignificant effects emerge for age, social class, education and religious identification, consistent with the current literature [12,46], with the exception of marginal effects in one of the four models for religious identification and for age.

Next, we tested whether one’s measured (self-reported) level of belief in God’s control moderates the effect of the primes inducing belief in God’s control (Models 2 and 4). Whereas some scholars find that religious cues affect political attitudes independently of one’s devoutness [13,26], it could be contended that strong belief in God’s control over the world may diminish the effect of the primes, due to de-sensitization to these stimuli, or alternatively may intensify the effect of the primes [39], as such individuals may more easily respond to subtle cues regarding God’s control. Models 2 and 4 in Table 2 examined the potential interactive effect of measured and manipulated belief in God’s control by adding two interaction terms to each of the models: an interaction between the question order manipulation and measured BGC, and an interaction between the essay manipulation and measured BGC. Results indicate that the question order manipulation was effective regardless of the participant’s reported level of BGC, as indicated by the high standard errors of the coefficients of the respective two interactive terms, for both outcome variables. Three of the four interaction terms yield insignificant coefficients, indicating that the manipulations were overall effective regardless of the participant’s reported level of BGC (as reported by [35]), with the exception of a marginally significant interaction for the essay prime in the expenditure model (p = .072). The S1 Fig plots the interactive effect of the essay manipulation and measured BGC on support for welfare state expenditure. The plot suggests that induced BGC increases support for welfare state expenditure for individuals believing in God’s control over the world (the thick gray line), but this effect wanes as measured BGC decreases.

All in all, the results of the first study provide evidence for the strengthening effect of BGC among Israeli Jews (H_2_). The subsequent studies are aimed at testing the generalizability of this result by varying the nature of the sample.

## Study 2

In study 1, we have observed that priming BGC strengthens WSA among Israeli Jews. Is this effect context-sensitive? In order to control for context effect, we replicated the experiment among Jews living in a substantially different political and cultural context, namely, the US, which is home to the largest concentration of Jews outside Israel [49]. The US is an interesting context for replication, due to its different economic discourse and since its political culture is characterized by a stronger relationship between religion and economic policy than that in Israel (see the discussion section below).

### Participants

Seventy-seven American Jews participated. The participants were recruited using a Qualtrics link that was distributed through virtual outlets in a method aimed at attaining a diversity of the sample with regard to age, sex, religiosity, social class, and geographical location. Specifically, the link to the experiment was distributed via the following outlets: Facebook groups of American alumni of Taglit (a popular program of travel to Israel for overseas Jewish youth; this pool is characterized by young age, geographical diversity, and relatively liberal religious affiliations [50]); mailing lists of Yeshiva University and Yeshivat Har-Etzion American Alumni (characterized by young age, east coast concentration, and Modern Orthodox affiliation); a Silver Spring, MD Jewish community mailing list (characterized by diverse age, geographically east coast, religious diversity skewed towards Orthodoxy); Facebook groups of diverse west coast religious institutions (diverse age, west coast concentration, and non-Orthodox affiliation); Facebook group of Pardes (learning program in Israel intended for American students) alumni (characterized by young age, geographically and religiously diverse); Amazon's Mechanical Turk (see Study 3 for details about the service).

As the descriptive statistics (see Table 3) show, diversity was largely attained. Still, the sample is relatively educated, and the vast majority of participants reside on the east coast (67 out of 77). Yet, this skewed geographical distribution resembles in some sense real-world demographics: firstly, as the Northeast region is the most Jewishly populated region in the US [51]. Secondly, it is a documented fact that American Jews have substantially higher educational attainment than the general American population [38].

**Table 3:** Descriptive statistics, American Jews sample (Study 2).

Variable	Mean	SD	Min	Max
Age	37.500	14.673	19	71
Male	0.519	0.503	0	1
Class	3.276	0.645	1	5
Religiosity	3.360	1.301	1	5
Ideology (conservative)	4.618	2.361	1	9
Education	5.355	0.934	2	7
BGC	4.447	1.816	1	7
Government responsibility for redistribution	2.623	1.278	1	5
Welfare state expenditure	3.295	0.945	1	5

### Ethics statement

This study was approved by the Institutional Review Board of the Political Science Department at the Hebrew University of Jerusalem and met all applicable standards for the ethics of experimentation and research integrity. Written informed consent was obtained from participants before they completed the questionnaire. All participants were 18 years of age or older.

### Procedure

Upon clicking on the distributed link, the participants were directed to a Qualtrics-powered online questionnaire. The questionnaire was essentially the English version of the questionnaire used in Study 1. The priming instrument was the English original of Laurin et al. [3], similar to the question order prime in Study 1, and the measures for the dependent variables were the same as in study 1.

Demographics. As in Study 1, we controlled for the following: For *religiosity* we used both Laurin et al.’s [3] BGC items employed as a prime, as well as Laurin et al.’s [3] original subjective religiosity scale: “How religious do you consider yourself? Please use this scale to indicate. One means ‘not at all religious’ and 5 means ‘very religious’” (a five-point scale, 1 = not at all religious, 5 = very religious), *age* (in years), *sex* (1 if male), *class* (5-point scale, 1 = low class to 5 = high class), *political orientation* (1–9, 9 = conservative), *education* (1–7, 1 = less than high school, 7 = more than a master’s degree). Table 3 reports descriptive statistics of the American Jewish sample.

### Results

**Fig 2:**
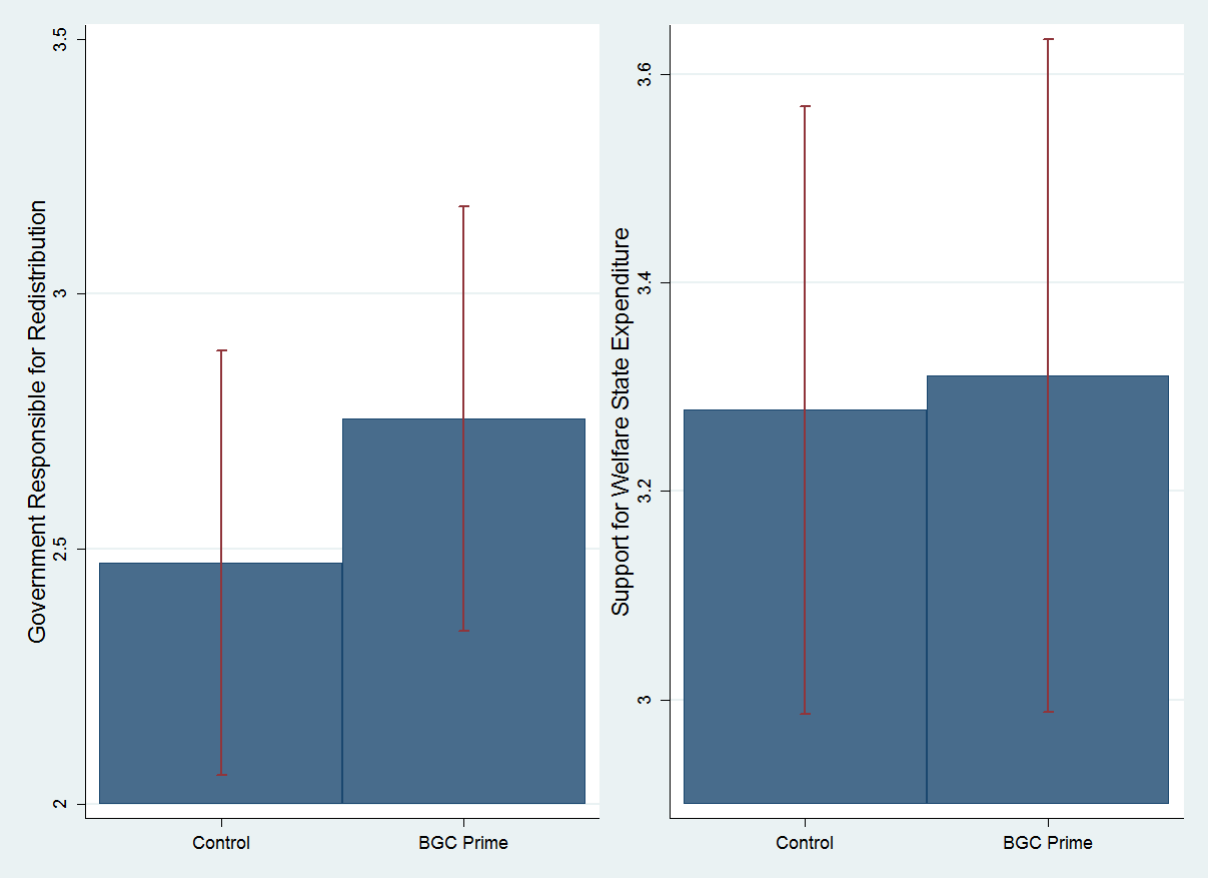
The Effect of the BGC Question Order Prime on Support for Redistribution and Support for Welfare State Expenditure among American Jews (Study 2). The graphs include 95% statistical significance bars.

The difference-of-means test for the two dependent variables, presented in Fig 2 below, suggests that the experimental condition increased one’s agreement that the government should take measures to reduce differences in income levels by about 7% of its range (_redistribution_ = 2.47 vs. 2.76 on a 1–5 scale, for the control and prime conditions, respectively), but that this effect is statistically insignificant, as indicated by a simple ANOVA (p_redistribution_ = .334). The prime did not affect support for welfare expenditures, as indicated by the similar means in the control and experimental conditions (_expenditure_ = 3.28 vs. 3.31 on a 1–5 scale, respectively) and the simple ANOVA test (p_expenditure_ = .879).

Models 1 and 4 in Table 4 test the effect of priming God’s control on both measures of welfare attitudes when controlling for demographics. To facilitate comparison, the controlled models are similar to the models in the Israeli Jewish sample, with slight adjustments of the measures (see the respective methods sections), and are identical to Laurin et al. [3], with an additional control for education.

**Table 4:** Effect of BGC on WSA among American Jews (Study 2).

	Government Responsible for Redistribution			Support for Welfare State Expenditure		
	1	2	3	4	5	6
BGC Prime	0.345+	-0.517	1.985*	-0.0665	-0.157	1.371+
	(0.202)	(0.586)	(0.941)	(0.158)	(0.450)	(0.741)
Age	0.00634	0.00753	0.00540	0.00583	0.00595	0.00500
	(0.00774)	(0.00792)	(0.00764)	(0.00598)	(0.00606)	(0.00584)
Male	-0.106	-0.0782	-0.0845	-0.0784	-0.0755	-0.0597
	(0.204)	(0.205)	(0.210)	(0.159)	(0.157)	(0.155)
Class	0.296+	0.311+	0.226	0.0324	0.0340	-0.0287
	(0.159)	(0.166)	(0.167)	(0.111)	(0.113)	(0.111)
Religiosity	0.173	0.176	0.172	0.107	0.108	0.106
	(0.119)	(0.118)	(0.121)	(0.0709)	(0.0712)	(0.0721)
Ideology (conservative)	-0.401**	-0.421**	-0.397**	-0.263**	-0.265**	-0.259**
	(0.0536)	(0.0518)	(0.0544)	(0.0430)	(0.0443)	(0.0412)
Education	0.180+	0.200+	0.349*	0.216*	0.218*	0.364**
	(0.105)	(0.108)	(0.153)	(0.0833)	(0.0859)	(0.104)
BGC (measured)	-0.184*	-0.308*	-0.179+	-0.187*	-0.200*	-0.182*
	(0.0897)	(0.118)	(0.0917)	(0.0706)	(0.0873)	(0.0721)
BGC X Prime		0.187			0.0197	
		(0.115)			(0.0967)	
Education X Prime			-0.308+			-0.270+
			(0.176)			(0.142)
Constant	2.429**	2.876**	1.741*	3.548**	3.595**	2.945**
	(0.708)	(0.688)	(0.803)	(0.449)	(0.500)	(0.487)
Observations	73	73	73	73	73	73
R-squared	0.613	0.626	0.624	0.609	0.609	0.625

Robust standard errors in parentheses

** p<0.01

*p<0.05

+ p<0.1

When partialling out the variance due to one’s demographics, the effect of induced BGC on agreement that the government is responsible for reducing inequality yields marginal statistical significance (p = .093), increasing this outcome variable by about 9% of its range. However, the effect of the prime remains insignificant for support for welfare expenditures (p = .676).

Next, we tested whether the effect of the prime is contingent on the reported level of belief in God’s control. Models 2 and 5 in Table 3 report the interactive effect of measured and manipulated BGC. As in Study 1, these models return a null result for the interactive terms, suggesting that the effect of induced BGC was not moderated by one’s level of belief in God’s control.

Compared to the findings in the Israeli sample, the effect of the question-order BGC prime is weaker and less robust for endorsement of government responsibility and does not emerge for support for expenditures. Still, the American Jewish sample differs from the Israeli Jewish sample in several respects. Most strikingly, compared to the Israeli Jewish sample (cf. Table 1), the American Jewish sample is much more educated. Thus, 4% in the American sample vs. 26% in the Israeli sample had a high school education or less, whereas 93% in the American sample had a college degree or more (of them 34% hold a master’s degree or more), vs. 34% in the Israeli sample (p_ttest_ = .000). Further, the American Jewish sample is older (_age_ = 37.50 vs. 26.02, p_ttest_ = .000), and more liberal (_ideology_ =. 452 “tending liberal” vs. .596, “tending right,” when both scales are normalized to vary 0–1; p_ttest_ = .001). There are no significant differences between the two samples in reported class, self-defined religiosity, or gender.

To examine the extent to which these differences between samples in education, age, and ideology may account for the different results, we specified interactions between each of the three variables and the prime.

Results show that both interactive terms of manipulated BGC and education yield marginally significant coefficients, as presented in Models 3 and 6 in Table 3 (p_redistribution_ = .085; p_expenditure_ = .062). As depicted in the S2 Fig, the effect of the prime is greatest for the lowest level of education, significantly increasing both measures of support for welfare (for education = 1: β_redistribution_ = 1.677, p_redistribution_ = .033; β_expenditure_ = 1.101, p_expenditure_ = .072), and the effect wanes or even reverses as the level of education increases (for education = 7: β_redistribution_ = -.171, p_redistribution_ = .645; β_expenditure_ = -.519, p_expenditure_ = .099). This post hoc finding suggests that the less educated are more amenable to the prime, such that the higher level of education in the sample may be at least partly responsible for the weaker results in the American Jewish sample. However, note that interaction with education did not yield statistical significance in the Jewish Israeli sample (p_redistribution_ = .273, .550; p_expenditure_ = .848, .318, for the question order and the essay prime, respectively) and the American Catholic sample (p_redistribution_ = .587; p_expenditure_ = .427). Thus, this result should be seen as tentative and should be further examined in future research (for further analysis of these results, see S1 Supporting Information).

With regard to the potential moderating role of education, a long-standing research in political psychology suggests that education affects amenability to political messages [52,53], facilitating people’s cognitive skills and political sophistication, and improving their ability to connect their ideological stances to the corresponding issue positions [54–57]. Accordingly, it was found that the more educated are better able to resist political messages with which they disagree, building on their ideology and information, whereas the less educated are less “principled” and more susceptible to priming [53,58–60].

In the same vein, previous studies show that among Americans belief in divine control is significantly lower among individuals who enjoy a high level of education and socio-demographic status [61], while their belief in personal control is significantly higher [62,63]. In addition, Jews are known to have a higher level of education on average [38]. Lower levels of BGC and high levels of belief in personal control might render individuals resilient to BGC priming, as it does not play a significant role in their cognitive apparatus. With regard to ideology and age, the interactions with the prime did not return statistical significance (ideology: p_redistribution_ = .695; p_expenditure_ = .644; age: p_redistribution_ = .627; p_expenditure_ = .062).

Moving to the control variables, conservative political ideology was associated with reduced support for welfare in both indicators. This result is in step with research on support for welfare in the United States, where conservative ideology is a central determinant of anti-welfare positions, supporting notions of individual responsibility while legitimating structural inequality and advancing pro-market attitudes [64]. According to the “self-interest” paradigm, lower classes and those who are economically vulnerable and thus more likely to receive welfare benefits tend to be more supportive of the welfare state [65]. Still, this sample yielded an unexpected post hoc result: that educational attainment and higher social class were positively associated with endorsement of government responsibility (p_redistribution_ = .093 and .068, respectively), and education was correlated with support for welfare expenditures as well (p_expenditure_ = .012). This seems to be a manifestation of the general trend of economic liberalism among wealthier American Jews [66]. Increased reported belief in God’s control was associated with decreased welfare support. Age, gender, and religious identification did not yield significant effects on welfare attitudes.

Overall, the null hypothesis for the influence of BGC on the American Jews sample can be cautiously and marginally rejected with respect to agreement that the government is responsible for redistribution, as per the strengthening hypothesis (H_2_), but not with respect to support for welfare state expenditures. The weaker results compared to the significant and positive effect among Israeli Jews could be partly due to differences in the sample characteristics, particularly with regard to levels of education, but could also be due to contextual differences, which suggest an ambivalent effect of BGC on support for the welfare state. Due to the tentative nature of this additional analysis, future research is needed to examine these expectations. The additional sample of American Catholics allows us to better understand whether the weaker results are mostly due to the particular sample or can be attributed to the different political context. If a weakening or a null effect for BGC is observed within the additional American sample, we will be able to cautiously attribute the differences from the Israeli sample at least in part to the political context. But if a clear strengthening effect is observed among American Catholics, we can cautiously attribute the weaker results to the sample.

## Study 3

Thus far, we have limited our investigation to one religious tradition–Judaism, varying its political context between Israel (Study 1) and the US (Study 2). We now aim to conduct the same experiment among American Catholics. This is especially important as our results thus far stand in contrast to those of Laurin et al. [3]: The latter showed that priming for the idea of God’s control decreased support for the institutions of law enforcement and punishment. Their sample was largely based on North American Christian university students. As a result, extending our investigation to a Christian sample would be an important test for the generalizability of the effect of priming belief in God’s control.

### Participants and procedure

We collected data for this study using participants recruited with Amazon’s Mechanical Turk (MTurk), an online crowd sourcing marketplace increasingly used in social scientific experimental research [67]. MTurk samples–while not as representative as the best national representative samples–usually have better demographic distributions than typical convenience samples. Indeed, MTurk data at least resemble “high quality” internet surveys, they have been used to replicate classic experimental findings [67,68], and results based on them have appeared in key journals (e.g., [69]).

Our participants were paid $0.50, which is consistent with standard rates on MTurk [70]. In order to ensure the quality of responses, we used the MTurk filtering mechanism to restrict the sample to those who achieved at least a 95% approval rate on at least 50 MTurk tasks. Eighty participants identifying themselves as Catholics were included in the sample. Materials were identical to those used in Study 2. Table 5 reports the descriptive statistics for the Catholic sample.

**Table 5:** Descriptive statistics, American Catholic sample (Study 3).

Variable	Mean	SD	Min	Max
Age	35.278	12.310	19	63
Male	0.613	0.490	0	1
Class	2.633	0.803	1	4
Religiosity	3.141	1.148	1	5
Ideology (conservative)	4.899	2.116	1	9
Education	4.150	1.244	1	6
BGC	4.612	1.568	1	7
Government responsibility for redistribution	3.063	1.266	1	5
Welfare state expenditure	3.347	0.844	1	5

### Ethics statement

This study was approved by the Institutional Review Board of the Political Science Department at the Hebrew University of Jerusalem and met all applicable standards for the ethics of experimentation and research integrity. Written informed consent was obtained from participants before they completed the questionnaire. All participants were 18 years of age or older.

### Results

**Fig 3:**
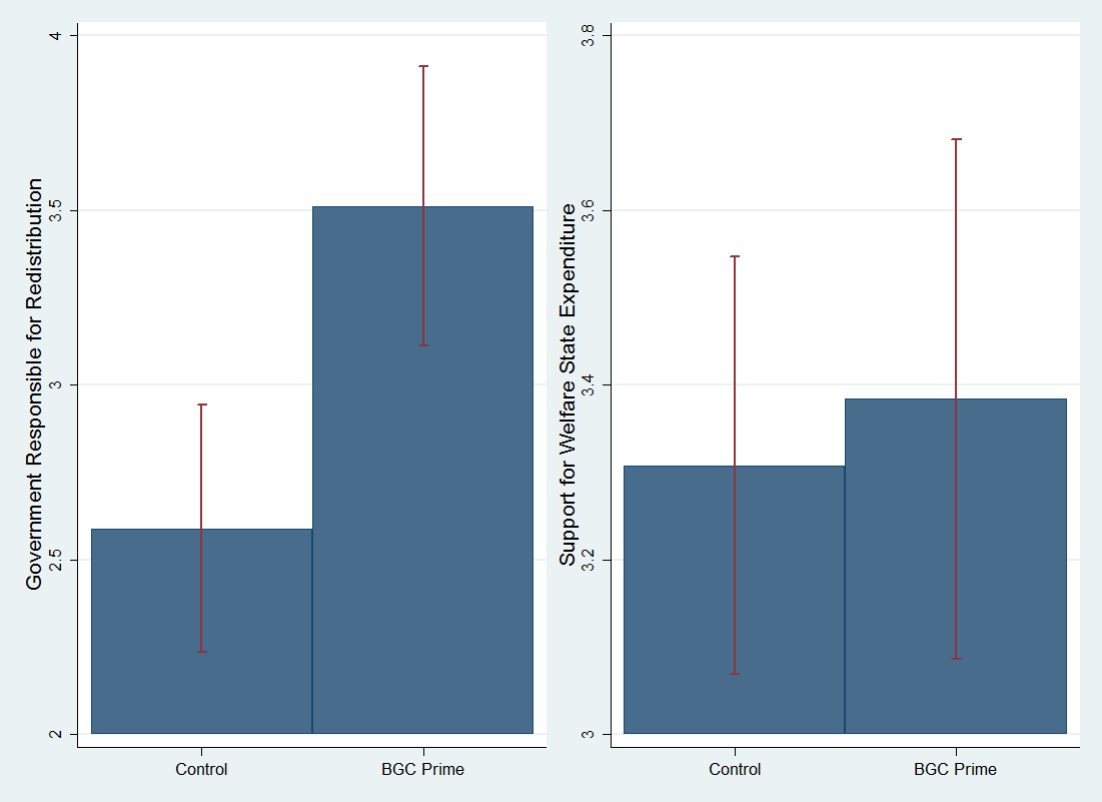
The Effect of the BGC Question Order Prime on Support for Redistribution and Support for Welfare State Expenditure among American Catholics (Study 3). The graphs include 95% statistical significance bars.

The difference-of-means test for the two dependent variables, presented in Fig 3 below, suggests that the experimental condition increased agreement that the government is responsible for redistribution by about 23% of its range (_redistribution_ = 2.59 vs. 3.51 on a 1–5 scale, for the control and prime conditions, respectively). This effect is statistically significant, as indicated by an ANOVA (p_redistribution_ = .001), supporting the strengthening hypothesis (H_2_). However, the prime did not affect support for expenditure, with overall similar means in the control and experimental conditions (_expenditure_ = 3.31 vs. 3.38 on a 1–5 scale, respectively) and the simple ANOVA test (p_expenditure_ = .688).

Table 6 presents the results of multivariate regression analysis for the effects of the priming in the Catholic sample, when controlling for background variables (Models 1 and 3). As in the difference-of-means analysis, results vary for the two outcome variables. Holding all else constant, manipulated belief in God’s control increased one’s agreement that the government is responsible for redistribution by 18% of its range, and this effect is statistically significant (p = .005). For welfare state expenditure, the effect of the prime is positive but statistically insignificant (p = .589).

**Table 6:** Effect of BGC on WSA among American Catholics (Study 3, Catholic sample).

	Government Responsible for Redistribution		Support for Welfare State Expenditure	
	1	2	3	4
BGC Prime	0.709**	1.297+	0.0840	1.125*
	(0.246)	(0.672)	(0.154)	(0.475)
Age	-0.0235*	-0.0229*	-0.00265	-0.00165
	(0.0104)	(0.0105)	(0.00699)	(0.00661)
Male	0.0311	0.0167	-0.135	-0.161
	(0.252)	(0.250)	(0.148)	(0.149)
Class	-0.0115	-0.0322	-0.198+	-0.234*
	(0.166)	(0.170)	(0.115)	(0.112)
Religiosity	0.405**	0.407**	0.0414	0.0447
	(0.152)	(0.150)	(0.101)	(0.0986)
Ideology (conservative)	-0.283**	-0.276**	-0.227**	-0.214**
	(0.0565)	(0.0563)	(0.0527)	(0.0494)
Education	-0.183+	-0.182+	-0.0471	-0.0445
	(0.0974)	(0.0973)	(0.0828)	(0.0779)
BGC (measured)	-0.200+	-0.144	0.0482	0.146+
	(0.108)	(0.129)	(0.0750)	(0.0828)
BGC X Prime		-0.124		-0.219*
		(0.130)		(0.0879)
Constant	5.328**	5.053**	4.921**	4.433**
	(0.684)	(0.735)	(0.548)	(0.545)
Observations	74	74	74	74
R-squared	0.457	0.462	0.385	0.427

Robust standard errors in parentheses

** p<0.01

* p<0.05

+ p<0.1

Next, we test whether one’s self-reported level of belief in God’s control moderates the effect of the primes inducing belief in God’s control by specifying an interaction term between the prime and measured BGC (Models 2 and 4). Results indicate that the experimental manipulation affected agreement with the idea that the government is responsible for redistribution regardless of the participant’s reported level of BGC, as indicated by the high standard errors of the coefficients of the respective interactive term in Model 2 (p = .345).

With respect to support for welfare expenditures, while the main effect of the prime did not reach statistical significance (cf. Model 3), it significantly interacted with measured BGC (p = .015). S3 Fig depicts this interactive effect for support for welfare state expenditures. As presented in Model 4 and in the S3 Fig, a positive effect of the prime is now evident for the low believers (β = .906, p = 0.024). Yet, this effect wanes and even slightly reverses itself among the high believers (β = -.409, p = 0.074). This phenomenon may be the result of a “ceiling effect”; that is, the extra awareness provided by the BGC prime makes a difference only for those who are less aware of it in their everyday lives, and not for those who are already aware of it without the priming.

Overall, the effect of the question-order BGC prime on endorsement of the idea of government responsibility is comparable to the results in the Israeli sample, but the results for support for welfare expenditures only emerge for low believers in God’s control. The differences between the two outcome variables are in step with findings from the Israeli Jewish sample, where the primes had greater impact on agreement with the government’s responsibility than on support for particular expenditures in return for a tax increase. Still, we were interested in comparing the characteristics of the two samples. Results from t-tests suggest that the American Catholic sample is older (p_ttest_ = .000), less educated (p_ttest_ = .001), of a lower self-reported class (p_ttest_ = .000), and more liberal (p_ttest_ = .005) than the Israeli Jewish sample (recall that ideology is defined differently in the two settings). The Catholic American sample is also significantly less educated on average than the Jewish American sample in Study 2 (pttest = .000), but does not significantly differ from the Jewish American sample in other background variables, including ideology (pttest = .437) and age (pttest = .308). To examine the extent to which these differences between the samples in age, education, class, and ideology may account for the different results, we specified interactions between each of the four variables and the BGC prime.

With regard to education, class, and age, none of the six interactions with the prime returned statistical significance (education: p_redistribution_ = .587; p_expenditure_ = .427; class: p_redistribution_ = .972; p_expenditure_ = .726; age: p_redistribution_ = .282; p_expenditure_ = .873). Finally, ideology did not significantly moderate the effect of the prime for endorsement of government responsibility (p_redistribution_ = .147), but its interaction yielded marginal statistical significance for support for welfare expenditures (p_expenditure_ = .096). As depicted in the S4 Fig, the effect of the BGC prime on support for welfare expenditures is positive and marginally significant for the most liberal participants (β = .606, p = .089), but this is reversed for the most conservative (β = -.461, p = .193). Accordingly, while we find that induced BGC increases attribution of responsibility to the government in matters of inequality, one’s willingness to pay more taxes depends on one’s political beliefs regarding whether tax increases are the right way of solving social problems. Thus, the effect of the BGC prime is moderated both by one’s level of BGC and one’s ideology.

Moving to the control variables, conservative political ideology was associated with reduced support for welfare in both indicators, as expected [64] and in accord with the results of Study 2. Note the inconsistent effect of ideology in the Israeli Jewish vs. the two American samples. Research on support for welfare in the United States typically reports a strong and robust effect of political ideology on welfare attitudes, since conservative ideology is taken as a central determinant of anti-welfare positions, supporting individual responsibility and structural inequality and advancing pro-market attitudes [64]. In contrast, Israeli politics is governed by attitudes on security and foreign policy issues, such that attributions of “left” and “right” ideology are determined by the extent of one’s support for peace and attitudes on the territories [71]. Economic and social issues are usually of relatively minor concern [47], and although right-wing ideology is overall associated with reduced support for welfare, public opinion on the security and economic dimensions is only weakly related [48]. This suggests that ideology in Israel, rooted in security attitudes, should be a weaker determinant of economic attitudes than in the American setting, as we indeed observed. As per the “self-interest” paradigm [65], both higher social class and greater educational attainment were related to decreased welfare support, although each of the two effects yielded statistical significance for one of the two outcome variables. Younger age and self-defined religiosity were both connected with overall agreement regarding the government’s responsibility, while gender did not yield a significant effect on welfare attitudes.

A comparison of the solid effect here and the weaker effect among American Jews might suggest that it is not the American context that dampened the effect. However, it could be the case that the documented liberal discourse of Catholic clergy [66] has led to a peculiar positive cognitive association between BGC and support for the welfare state. However, we could not verify this suggestion in the current research.

## General Discussion

The relationship between religion and pro-sociality in general, and religion and support for the welfare state in particular, has been shown to be complex and multifaceted [10,12,23–25]. The current study examines the influence of a specific belief–belief in God's control–on approval for the idea of assisting others through the welfare state. We presented results from a series of three experimental studies, aimed at investigating the nature of this influence. This investigation had two main goals: Firstly, to test competing hypotheses regarding the direction of the effect in general. Secondly, within a context-sensitive framework, to test the generalizability of the results across political contexts (Israel vs. the US) and religious traditions (Judaism vs. Catholicism). We now turn to discussing our findings and their contributions to the extant literature. We then conclude by discussing methodological issues as well as possible future directions in the field.

### Direction of influence: the strengthening effect of BGC on welfare state attitudes

Basing ourselves on previous literature, we formed two conflicting hypotheses regarding the direction of the effect of BGC on support for the welfare state: one that anticipates a weakening effect (H_1_) and one that anticipates a strengthening effect (H_2_). In general, the trend in the various studies points to a greater validity of H_2_: In all three samples, we exhibited at least a marginally significant strengthening influence for induced BGC on the support for government redistribution of income, using two priming instruments (a question order prime in all three samples, and an additional essay prime in the Israeli Jews sample).

Although we did not observe a weakening effect in any of the samples, outcome variables, or primes, the strengthening effect for the welfare state expenditure outcome variable and the American Jews sample were somewhat weaker. However, we think these deviations from the strengthening trend could be explained. Firstly, the expenditure outcome variable is more demanding as participants are explicitly asked to consider the increased tax burden. Secondly, the American Jewish sample was much more educated than other samples were. Indeed, moderation analysis showed a strengthening effect on the less-educated.

This could be explained by previous research that gives evidence that priming is less effective among the more-educated [53,58–60] and that BGC has less existential relevance for them [61–63] (although this post hoc observation deserves further research). Based on these results and considerations, it seems that the findings suit the strengthening hypothesis (H_2_) better than the weakening hypothesis (H_1_).

As the conflicting expectations were both based on existing theory and evidence, our empirical findings reinforce some previous ones, while challenging others: first, the evidence for the strengthening effect is consistent with previous evidence on the positive association between some aspects of religious belief and support for the welfare state. Arikan and Ben-Nun Bloom [10] concluded, based on comparative-correlative analysis, that while the religious social dimension tends to decrease support for welfare state measures, the belief dimension can also have positive effects (however, the overall effect of the belief dimension is negative, because of the much stronger association between religious belief and conservative political orientation, which is usually characterized by lower WSA). In addition, our study reinforces claims that religion might foster demand for the welfare state, caused by lower levels of a sense of personal control induced by BGC [9].

However, these findings are in contrast with previous evidence that posits a weakening effect of BGC on the endorsement of interventionist political institutions. Building on a theoretical model that predicts substitution relations between God and the government [1,5], few experimental studies have shown that priming God’s control tends to decrease support for a strong government in general [2] or for judicial punishment [3], with the latter using the same prime instrument utilized in our study. We suggest two explanations for this disagreement in the findings.

First, the divergence in results could be attributed to the difference in the dependent variable. While the aforementioned studies have investigated the effect of BGC on support for strong government in general [2] as well as on judicial punishment [3], our research focuses on welfare attitudes. As the welfare state may be perceived as an institution associated with religious charity obligations common to many religious traditions, the competition between God and political institutions is less pronounced. Therefore, the “complementary relations” model may be more fitting than the “substitution relations” model.

Secondly, at the technical level, we argue that our sampling methodology engenders greater internal validity. A limitation acknowledged by Laurin et al. ([3], p. 3279) is that their samples were limited to North American participants and pooled together different religious traditions, including Islam and Eastern traditions. In contrast, we employed two different primes in two political contexts for two religious traditions.

Still, both religiosity and welfare support are multi-dimensional. Other measures and elements of welfare attitudes may yield divergent results. For religiosity, we focused on a particular type of belief, although current work suggests that different facets of religiosity sometimes yield different, and even contrasting, results [26,35].

The positive effect of belief in God’s control on support for welfare that emerged in this study is in contrast to the Marxist tradition, according to which such beliefs necessarily promote social conservatism. Since historically this Marxist view has driven a wedge between religiosity and left-wing parties in many political contexts [43], our findings may have practical implications for both religious groups and supporters of left-wing economics. First, they may facilitate recognition of a possible common ground. Secondly, they suggest the possibility that messages that include BGC in a religious discourse might provide a fertile ground for political mobilization in favor of left-wing economics. As previously suggested in the literature, priming experiments may emulate the effect of political cues [35,72]. Consequently, political and religious leaders willing to strengthen welfare attitudes might want to incorporate BGC into their public discourse.

### Sensitivity to context

Does it make more sense to study religion as a universal phenomenon and consider those ideas that are common to all religions, above and beyond the context (e.g., [30,36]), or to focus on different religions in their particularity (e.g., [37])? In recent years a synthesis of these two positions has emerged, according to which “it seems reasonable to presume that there should be both universals and cultural specifics to religion and individual religiosity” ([73], pp. 1320–1321), meaning that both overall trends and differences between religious traditions and political contexts would be visible.

Our results appear to be in line with the latter approach. While we largely replicated the positive effect of BGC on WSA across two political settings and two religions, the strength of the effects varied between samples. Ranking the samples by their strength, we observed the strongest effect among Israeli Jews (Study 1), followed by American Catholics (Study 3), and then American Jews (Study 2). Still, we observed a strengthening effect in all cases, including for one of the outcome variables and for subsets of the sample among American Jews, while a weakening effect did not emerge in any of the analyses.

The weaker effect for American Jews (compared with the Israeli Jews) could be partly due to differences in political context. First, empirical investigation of the US population has shown that in the US, a majority endorses “natural” market outcomes as fair [6,74]. In contrast, Israelis tend to be more skeptical than Americans about market outcomes. Although Israel’s economic discourse has shifted in the neo-liberal direction in the last 25 years [75], it still has deep roots in European-style welfare state discourse. In fact, attitudinal support for income redistribution in Israel is among the highest in the Western world [44,76]. It is claimed that Weber’s famous theory of the Protestant Ethic accounts for American exceptionalism in this regard, such that the Protestant religious origins of the US’s culture led to a cultural tendency to believe that “market” economic outcomes are fair [6,31]. In this view, God’s providence assigns economic success to certain individuals, thus fostering belief in deservingness and weaker support for the welfare state.

Secondly, the tie between religion and welfare attitudes is stronger in the US than it is in Israel. It has been noted that many major sociopolitical movements in modern US history have had a religious orientation, from the left-wing Social Gospel, abolitionism, and the civil rights movement, to the right-wing Tea Party movement [66]. Whereas religion plays an important role in shaping Americans’ economic ideology, it seems that in Israel, religion, although an important factor in national politics, is less influential in the formation of economic preferences. In Israel, religious values are strongly connected to the security and foreign policy issues that largely drive Israeli politics [71], and rarely linked to economic issues, which are usually of more minor concern [47]. As a result, Israeli Jews do not typically connect their religious beliefs to economic attitudes, and are even less concerned with economic issues than secular Israeli Jews. For example, a recent poll has found that while a majority of non-religious Israelis sees economic issues as an important factor in their electoral voting decisions, only about one-third of the religious find them important [76]. Given this difference between American and Israeli political discourse, the effect of experimentally induced belief in God’s control may be more pronounced in Israel, where participants are less exposed to religious argumentations regarding economic policy.

Still, when holding the context constant (US) and varying the religious tradition (American Catholics vs. American Jews), the strengthening effect emerging for American Catholics was stronger than the effect among American Jews. Morris [77] distinguishes between religions of *assent* and *descent*. “Communities of assent are based on strategies of successful rhetorical persuasion, designed to lead to assent to a body of shared truths, or values” (p. 239). Descent-based religious communities, by contrast, are those to which a person normally belongs by virtue of biological descent. While Judaism is an example of a descent religion—every person born to a religious Jewish mother is considered by Jewish law as a Jew—Christianity relies on belief. Cohen [78] shows that, not just doctrinally but also demographically, belief is a more prevalent and influential component of their religion for Catholics than for Jews, which may partly account for the greater effect of BGC among Catholics, when holding constant the context.

Overall, we cannot clearly infer from the results whether the weaker effect among American-Jews is due to contextual effects, to particular characteristics of the sample (such as their higher level of education), or to a real ambivalence in the effect of BGC on support for the welfare state. The strengthening effect observed for American Catholics could supply tentative reinforcement that it is not the American context that weakens the effect of BGC. However, it still could be argued as being due to some peculiarity of American Catholicism since the documented liberal discourse of American Catholic clergy [66] seems liable to lead to a positive association between BGC and support for the welfare state. Future research could test this suggestion.

### Independence of primed and measured BGC

Whereas some scholars find that religious cues affect political attitudes independently of one’s devoutness [26,35], it could be contended that strong belief in God’s control over the world may diminish the effect of the primes, due to de-sensitization to these stimuli, or alternatively intensify the effect of the primes, as such individuals may more easily respond to subtle cues regarding God’s control [39].

In the current study, the positive effect of priming belief in God’s control was largely independent of the reported level of belief. Furthermore, among the only two exceptions (out of eight models), the moderation effect was inconsistent: among Israeli Jews (Study 1), the BGC prime increased welfare state expenditure for high believers (for one of the two primes), while in the American Catholic sample (Study 3) the BGC prime increased welfare state expenditure for low believers.

Why does religious priming make a difference for people who report a low level of belief? A possible explanation for this phenomenon is that even after the secularization processes of modernity, religion is still an important part of both contemporary American and Israeli culture, making religious beliefs, such as the idea of God’s control and its implications, a meaningful cultural reference [3,28].

### Concerns over priming methodology

In recent years, prominent scholars have expressed concerns about the reliability of priming methodology. Alongside the acknowledgment of the need for caution, we note the following reasons for a decreased concern regarding the use of priming in the current studies. First, a large part of the criticism concerns the habit in psychology of conceptual replication of experiments, which, unlike literal replication, changes the priming instrument across studies [79]. Our study, however, holds constant a priming instrument (the question-order prime) among the different samples. In addition, the prime employed in all three studies is importantly the same as the one used in Laurin et al.’s experiments on the effects of BGC on judicial punishment [3], which we aimed to generalize to support for welfare state. Another potential concern involves the possibility of experimenters’ expectations influencing the results [80]. Our study, however, relies mostly on minimal participation by the experimenters (or none at all), as an overwhelming majority of the participants were allocated to an experimental condition by a Qualtrics-powered virtual link. The participants who filled in a pen-and-paper version of the questionnaire comprise about 25% of the respondents in Study 1. Supplementary analysis suggests that the results reported for Study 1 hold even when those participants are dropped from the statistical analysis (question order prime: β_redistribution_ = .980, p = .001; β_expenditure_ = .392, p = .031; Essay prime: β_redistribution_ = .721, p = .015; β_expenditure_ = .297, p = .150).

### Future directions

Future studies can extend the current framework in several ways. First, belief in God’s control has thus far been investigated as a driver of lesser support for a strong government in general [2] and for judicial punishment in particular [3]. This study examines its effect on welfare attitudes, and shows different results from research on other policy domains. This suggests a domain specificity, such that belief in God’s control yields differential results for different political institutions. Future studies should examine additional dependent variables in order to shed more light on the complex nature of BGC.

Secondly, with regard to the sphere of religiosity and the welfare state, it could be valuable to experimentally investigate the effects of other specific beliefs, apart from God’s control. This could include, for example, belief in a benevolent God, which was found to increase personal benevolence in the lab (e.g., [81]), though its effect on support for the welfare state has not yet been tested. In addition, it would be interesting to test the influence of other dimensions of religiosity, other than belief, such as participation in a religious community. This behavioral dimension was found to decrease support for the welfare state in a comparative-correlative analysis, but has yet to be tested in experimental design [4,10].

Further, a common limitation in social psychology research is its vulnerability to “demand characteristics.” This is known to be especially significant with regard to the relationship between religion and pro-sociality, when the relationship is investigated using explicit, self-reported measures that raise impression-management considerations [17]. A useful complement to our study would be to replicate it with behavioral markers, such as economic games that emulate real-world decisions regarding welfare-state issues; for instance, games involving budgetary decisions (e.g. [82]).

## Supporting Information

S1 Appendix Essay priming (Study 1, English translation). (DOCX)

S1 Dataset Supporting Data for Studies 1–3. (XLSX)

S1 Fig The Interactive Effect of Measured and Manipulated BGC on Support for Welfare State Expenditure among Israeli Jews (Study 1). The graph includes predictive margins with 95% confidence intervals.
(TIF)

S2 Fig The Interactive Effect of Education and Manipulated BGC on Support for Welfare State Expenditure among American Jews (Study 2). The graphs include predictive margins with 95% confidence intervals.
(TIF)

S3 Fig The Interactive Effect of Measured and Manipulated BGC on Support for Welfare State Expenditure among American Catholics (Study 3). The graph includes predictive margins with 95% confidence intervals.
(TIF)

S4 Fig The Interactive Effect of Ideology and Manipulated BGC on Support for Welfare State Expenditure among American Catholics (Study 3). The graph includes predictive margins with 95% confidence intervals.
(TIF)

S1 File The Interactive Effect of Education and BGC Primes on WSA. (DOCX)
