# Supplementary material for: God and the Welfare State - Substitutes or Complements? An Experimental Test of the Effect of Belief in God's Control
Source: PLoS One. 2015 Jun 10;10(6):e0128858. doi: 10.1371/journal.pone.0128858 (PMC4463850; doi:10.1371/journal.pone.0128858)
Supplement: S1 Appendix — (DOCX) [file pone.0128858.s001.docx]

S1 Appendix: Essay priming (Study 1, English translation)

Please read the passage below carefully and answer the questions that follow.

God’s providence encounters us in all kinds of hidden ways. It’s true that most of the time it seems that the world simply goes on and things happen naturally. But at other times, we recognize “sparks” of a feeling that there is someone up above who has a plan for us. All kinds of things happen, all kinds of coincidences, that make it seem like someone is hinting to us that there is hidden intervention by someone who is making sure that those things happen. Although in the modern secular context it is argued that the routine way of the world shows that there is no divine providence, they ignore the possibility that God’s intervention in the world doesn’t have to be via obvious miracles that openly change the laws of nature or social behavior. God is sophisticated enough to use changes like the “butterfly effect,” in which a small change in one place eventually causes the desired intervention in another place.

The importance of this belief in God’s providence is to ensure the rule of God in the world, and his ability to implement his plan for us - even if we don’t always understand this plan due to our limited ability to comprehend it.

If we take these feelings and insights seriously, they can teach us that human beings are not the ones who run the world at the end of the day. We can recall the words of our sages of blessed memory, who said: “A man does not lift his finger below, unless it was declared above that he would do so” (Babylonian Talmud, Tractate Hulin). In other words, everything that happens in the world is done through the power of heavenly consent. The one who truly runs the world is the hidden hand of God’s providence. God is the King of the universe.

Please summarize the passage that you read in a few sentences.

__________________________________________________________________________________________________________________________________________
