# Supplementary figures and images for: God and the Welfare State - Substitutes or Complements? An Experimental Test of the Effect of Belief in God's Control
Source: PLoS One. 2015 Jun 10;10(6):e0128858. doi: 10.1371/journal.pone.0128858 (PMC4463850; doi:10.1371/journal.pone.0128858)

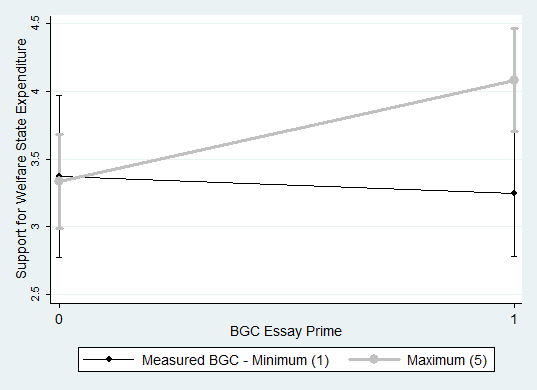

Supplement: S1 Fig — The graph includes predictive margins with 95% confidence intervals. (TIF) [file pone.0128858.s003.tif]

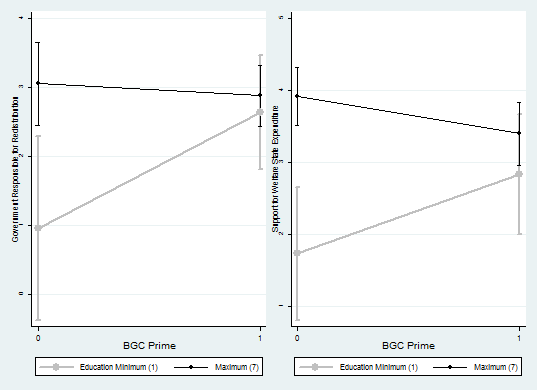

Supplement: S2 Fig — The graphs include predictive margins with 95% confidence intervals. (TIF) [file pone.0128858.s004.tif]

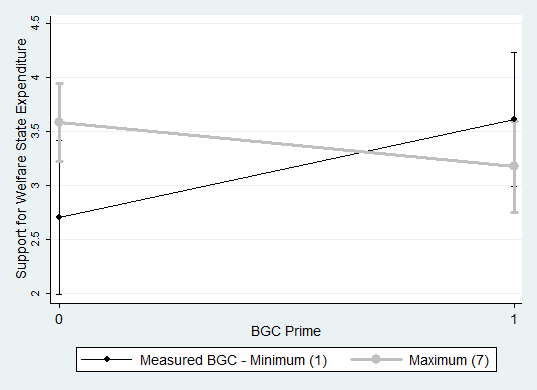

Supplement: S3 Fig — The graph includes predictive margins with 95% confidence intervals. (TIF) [file pone.0128858.s005.tif]

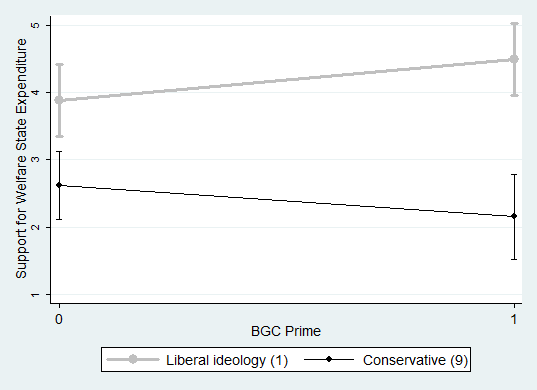

Supplement: S4 Fig — The graph includes predictive margins with 95% confidence intervals. (TIF) [file pone.0128858.s006.tif]
