# Supplementary material for: God and the Welfare State - Substitutes or Complements? An Experimental Test of the Effect of Belief in God's Control
Source: PLoS One. 2015 Jun 10;10(6):e0128858. doi: 10.1371/journal.pone.0128858 (PMC4463850; doi:10.1371/journal.pone.0128858)
Supplement: S1 File — (DOCX) [file pone.0128858.s007.docx]

**S1 Supporting Information. The Interactive Effect of Education and BGC Primes on WSA**

Table S1: The Interactive Effect of Education and BGC Primes on WSA

|  | **Israeli Jews** | | **American Jews** | | **American Catholics** | |
| --- | --- | --- | --- | --- | --- | --- |
|  | Government Responsible | Support Welfare | Government Responsible | Support Welfare | Government Responsible | Support Welfare |
|  |  |  |  |  |  |  |
| BGC Prime | 1.908+ | 0.489 | 1.985* | 1.371+ | 1.151 | 0.534 |
|  | (0.968) | (0.458) | (0.941) | (0.741) | (0.818) | (0.588) |
| BGC Prime 2 (Essay) | 1.331 | -0.141 | - | - | - | - |
|  | (0.960) | (0.574) |  |  |  |  |
| Age | -0.0117 | 0.00803 | 0.00540 | 0.00500 | -0.0230* | -0.00218 |
|  | (0.0189) | (0.00640) | (0.00764) | (0.00584) | (0.0107) | (0.00697) |
| Male | -0.641** | -0.394** | -0.0845 | -0.0597 | 0.0539 | -0.112 |
|  | (0.230) | (0.144) | (0.210) | (0.155) | (0.255) | (0.143) |
| Class | -0.148 | -0.0531 | 0.226 | -0.0287 | -0.00214 | -0.188 |
|  | (0.144) | (0.0715) | (0.167) | (0.111) | (0.167) | (0.116) |
| Religiosity | -0.168+ | -0.0682 | 0.172 | 0.106 | 0.400* | 0.0369 |
|  | (0.0907) | (0.0488) | (0.121) | (0.0721) | (0.154) | (0.0984) |
| Ideology (conservative) | -0.136* | -0.0547 | -0.397** | -0.259** | -0.278** | -0.222** |
|  | (0.0606) | (0.0389) | (0.0544) | (0.0412) | (0.0558) | (0.0503) |
| Education | 0.113 | -0.0897 | 0.349* | 0.364** | -0.140 | -0.00306 |
|  | (0.171) | (0.0899) | (0.153) | (0.104) | (0.108) | (0.0949) |
| BGC (measured) | 0.148 | 0.0812 | -0.179+ | -0.182* | -0.202+ | 0.0461 |
|  | (0.115) | (0.0658) | (0.0917) | (0.0721) | (0.110) | (0.0734) |
| Education X Prime | -0.222 | -0.0204 | -0.308+ | -0.270+ | -0.107 | -0.109 |
|  | (0.201) | (0.106) | (0.176) | (0.142) | (0.195) | (0.136) |
| Education X Prime 2 | -0.120 | 0.122 | - | - | - | - |
|  | (0.199) | (0.121) |  |  |  |  |
| Constant | 4.323** | 4.071** | 1.741* | 2.945** | 5.109** | 4.698** |
|  | (1.048) | (0.447) | (0.803) | (0.487) | (0.789) | (0.609) |
|  |  |  |  |  |  |  |
| Observations | 102 | 100 | 73 | 73 | 74 | 74 |
| R-squared | 0.300 | 0.273 | 0.624 | 0.625 | 0.459 | 0.391 |

Robust standard errors in parentheses; ** p<0.01, * p<0.05, + p<0.1

To examine the consistency of the moderation effect of education which was found among American Jews, we specified interactions between education and the prime in the other two samples as well. To facilitate interpretation of the conditional relationships, Figure S1 presents the effect of the BGC prime on predicted levels of endorsement of government responsibility (left-hand side panels) and support for expenditures (right-hand side panels) for the minimum and maximum levels of education.

The interaction with education returned a marginally significant result in both outcome variables in the American Jewish sample (p_redistribution_ =.085; p_expenditure_=.062), but did not yield statistical significance in the Jewish Israeli sample (p_redistribution_=.273, .550; p_expenditure_=.848, .318, for the question order and the essay prime, respectively) and the American Catholic sample (p_redistribution_ =.587; p_expenditure_=.427).

Figure S1: The Interactive Effect of Education and BGC Primes on WSA

The graphs include predictive margins with 95% confidence intervals.

Still, given the vast differences in the level of education between the American Jewish sample and the other two samples, we were interested in examining the general trends of these interactions. Looking at the figure above, the effect of the BGC prime for the minimum level of education, represented by the thick gray line in the panels, is positive in all three samples and across the two outcome variables, while the effect of the BGC prime for the maximum level of education, represented by the thinner black line, is milder, flat or even slightly reverses.
